# Cerebrovascular reactivity measured in awake mice using diffuse correlation spectroscopy

**DOI:** 10.1117/1.NPh.8.1.015007

**Published:** 2021-03-01

**Authors:** Rowan O. Brothers, Nir Atlas, Kyle R. Cowdrick, Erin M. Buckley

**Affiliations:** a Emory University and Georgia Institute of Technology, Wallace H. Coulter Department of Biomedical Engineering, Atlanta, Georgia, United States; b Emory University and Children’s Healthcare of Atlanta, Division of Critical Care Medicine, Department of Pediatrics, Atlanta, Georgia, United States; c Emory University School of Medicine, Department of Pediatrics, Atlanta, Georgia, United States; d Children’s Healthcare of Atlanta, Children’s Research Scholar, Atlanta, Georgia, United States

**Keywords:** cerebrovascular reactivity, diffuse correlation spectroscopy, acetazolamide, mice, cerebral blood flow

## Abstract

**Significance:** Cerebrovascular reactivity (CVR), defined as the ability of the cerebral vasculature to dilate or constrict in response to a vasoactive stimulus, is an important indicator of the brain’s vascular health. However, mechanisms of cerebrovascular dysregulation are poorly understood, and no effective treatment strategies for impaired CVR exist. Preclinical murine models provide an excellent platform for interrogating mechanisms underlying CVR dysregulation and determining novel therapeutics that restore impaired CVR. However, quantification of CVR in mice is challenging.

**Aim:** We present means of assessing CVR in awake mice using intraperitoneal injection of acetazolamide (ACZ) combined with continuous monitoring of cerebral blood flow.

**Approach:** Measurements of cerebral blood flow were made with a minimally invasive diffuse correlation spectroscopy sensor that was secured to an optical window glued to the intact skull. Two source–detector separations (3 and 4.5 mm) per hemisphere were used to probe different depths. CVR was quantified as the relative increase in blood flow due to ACZ. CVR was assessed once daily for 5 days in 5 mice.

**Results:** We found that CVR and the response half-time were remarkably similar across hemispheres and across 3- versus 4.5-mm separations, suggesting a homogenous, whole brain response to ACZ. Mean(std) intra- and intermouse coefficients of variations were 15(9)% and 19(10)%, respectively, for global CVR and 24(15)% and 27(11)%, respectively, for global response half-time.

**Conclusion:** In sum, we report a repeatable method of measuring CVR in free-behaving mice which can be used to screen for impairments with disease and to track changes in CVR with therapeutic interventions.

## 1 Introduction

Cerebrovascular reactivity (CVR), defined as the ability of the cerebral vasculature to dilate or constrict in response to a vasoactive stimulus, is an important indicator of the brain’s vascular health.^1^ Impaired CVR is present in numerous disease states, including cerebrovascular disease,^2^^–^^8^ stroke,^9^^–^^12^ cardiovascular disease,^13^^,^^14^ cardiac arrest,^15^ traumatic brain injury,^16^^–^^19^ diabetes,^20^ and sleep apnea.^21^ Moreover, several studies have suggested that impaired CVR may serve as a prognostic biomarker of functional outcome.^22^^–^^25^ Despite mounting evidence for the relevance of CVR as a potential disease biomarker, mechanisms of cerebrovascular dysregulation are poorly understood, and effective treatment strategies for impaired CVR are lacking.

Preclinical murine models provide an excellent platform for interrogating mechanisms underlying vascular dysregulation and determining novel therapeutics that restore impaired CVR. However, quantification of CVR in mice is challenging. Common techniques to assess CVR include intravenous injection of the potent vasodilator acetazolamide (ACZ) and induction of hypercapnia via inhalation of a high-concentration (typically 5% to 6%) carbon dioxide gas mixture. For both of these techniques, cerebral blood flow is monitored before and after intervention, and CVR is defined as the relative percent change in blood flow from preintervention levels normalized to either the dose of ACZ or to the change in arterial partial pressure of carbon dioxide. Although these assessments are routinely used in humans, delivery of the vasoactive stimulus and reliable monitoring of the stimulus response is more challenging in mice. In the case of ACZ, although intravenous tail vein injection is possible, longitudinal assessment of CVR with this approach is challenging because repeated tail vein injections without the use of a catheter cause inconsistent drug delivery and increase risk of infection and vein collapse.^26^ Alternatively, hypercapnia requires anesthesia for gas delivery in mice, which can induce significant confounding effects on the cerebrovasculature that lead to errors in the estimation of CVR.^27^^–^^32^ Moreover, monitoring the amount of carbon dioxide that has reached the blood stream to accurately estimate CVR during hypercapnia requires either invasive insertion of an arterial catheter to sample blood or intubation to measure end tidal carbon dioxide concentrations. Finally, both interventions to assess CVR (ACZ/hypercapnia) require quantification of cerebral blood flow, which is non-trivial in mice due to their small size. Although a handful of techniques exist to measure CBF in mice, e.g., autoradiography, perfusion magnetic resonance imaging, laser Doppler flowmetry, laser speckle contrast imaging, and microultrasound, these techniques typically involve invasive surgery, anesthesia, and/or manual restraint.^33^ In sum, challenges in both stimulus delivery and monitoring response to the stimulus make CVR measurements in murine models technically challenging, time consuming, and preclude longitudinal assessment.

Herein, we present a novel means of assessing CVR in mice that overcomes several of these technical limitations and enables longitudinal monitoring of CVR in awake, free behaving animals. In this approach, mice are given ACZ intraperitoneally, and the cerebral blood flow response is continuously monitored with diffuse correlation spectroscopy (DCS) using a minimally invasive sensor that is secured to the intact skull. Given the relative ease of both intraperitoneal injections and assessment of blood flow with DCS, multiple longitudinal measurements of CVR can easily be acquired. In this work, we characterize average CVR in healthy mice, and we quantify the spatial and temporal heterogeneity of the CVR response.

## 2 Methods

Five 3- to 4-month-old male C57BL/6 mice were used. Measurements of resting state cerebral blood flow and CVR were assessed once daily for 5 days at the same time each day. All animal procedures were approved by Emory University Institutional Animal Care and Use Committee and followed the NIH Guidelines for the Care and Use of Laboratory Animals.

### 2.1 Animal Preparation

To enable continuous monitoring of cerebral blood flow with DCS, a laser cut clear acrylic optical window (Part No. 8560K174, McMaster-Carr) was secured to the intact skull at least 3 days prior to the start of the study. To implant the window, mice were anesthetized with 1% to 2% isoflurane, a midline incision was made that extended from between the ears to behind the eyes, fascia was removed, and a thin (∼0.5  mm) layer of dental cement (C&B-Metabond, Parkell Inc., Edgewood, NY, USA) was employed to secure the optical window to the skull. The entire implantation procedure took ∼45  min from induction to recovery from anesthesia.

**Fig. 1:**
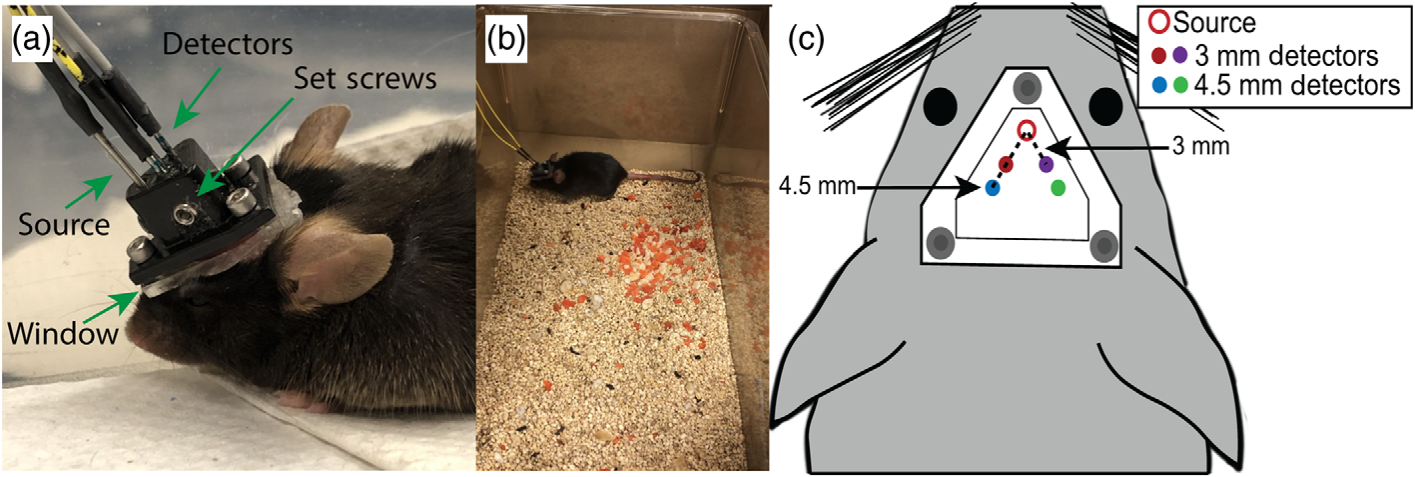
Measurements of cerebral blood flow in awake mice. (a) For longitudinal measurements of cerebral blood flow, mice were briefly restrained and a custom-made, lightweight optical sensor was secured to a clear acrylic window that was attached to the intact skull. (b) Measurements were made while the animal was allowed to ambulate freely in an empty cage. (c) Diagram of the acrylic window and optical sensor positioning on the mouse head. Gray circles denote position of the screws used to secure the optical sensor to the window. One source (open circle) and four detector fibers (closed circles) were arranged within the optical sensor to enable bilateral assessment of blood flow at source–detector separations of 3 and 4.5 mm (Video S1, MP4, 10 MB [URL: https://doi.org/10.1117/1.NPh.8.1.015007.1]).

Following a 3-day recovery period, mice were acclimated to the experimental protocol by securing a custom-made, lightweight DCS sensor to the optical window. To position the sensor, mice were briefly manually restrained, and the optical sensor was secured to the optical window with three screws [Fig. 1(a)]. Once secured, mice were placed in an empty cage and allowed to ambulate freely for 10 to 20 min [Fig. 1(b) and Video S1], at which time the sensor was removed and the mice were returned to their cage. This acclimation process was repeated once daily for 3 days.

### 2.2 Assessment of Cerebral Blood Flow and Cerebrovascular Reactivity

Measurements of cerebral blood flow were made with DCS.^34^^–^^36^ In DCS, a coherent near-infrared source is used to illuminate the tissue surface. A detector is placed at a fixed distance from the source to detect light that has multiply-scattered through the tissue. Scattering off moving red blood cells causes the detected light intensity to fluctuate with time. Correlation diffusion theory is used to relate these intensity fluctuations to blood flow index (BFI) within the underlying tissue. Although the units of BFI (${\mathrm{mm}}^{2}/s$) are not the traditional units of flow (mL/min/100  g), numerous validation studies,^37^ including a recent study in mice,^33^ have shown that BFI is correlated with blood flow measured by other perfusion modalities, including laser Doppler flowmetry,^38^^,^^39^ color Doppler ultrasound, and transcranial Doppler ultrasound,^38^^,^^40^^–^^43^ perfusion magnetic resonance imaging,^33^^,^^38^^,^^41^^,^^44^^,^^45^ Xenon-CT,^46^ near-infrared spectroscopy,^47^ and fluorescent microspheres.^48^

The DCS instrument employed herein consisted of an 852-nm long coherence-length laser (iBeam smart, TOPTICA Photonics, Farmington, NY), an array of four photon counting avalanche photodiodes (SPCM-AQ4C-IO, Perkin-Elmer, Quebec, Canada), and a hardware autocorrelator board (Flex05-8ch,^49^ NJ).^33^^,^^50^ The animal interface for the device consisted of a custom 3D printed optical sensor with a 400-μm multimode source fiber (FT-400-EMT, Thorlabs Inc., Newton, NJ, USA) and four single-mode detector fibers (780HP, Thorlabs Inc., Newton, NJ, USA) spaced 3 and 4.5 mm from the source [Figs. 1(a) and 1(c)]. These separations were chosen to ensure sensitivity to cortical tissue.^51^ Set screws on the sides of the sensor were used to secure all fibers tightly in place [Fig. 1(a)]. Data were acquired from all detectors simultaneously at a rate of 1 Hz.

The measured intensity autocorrelation function at each time t and source–detector separation r was fit to the semi-infinite homogeneous solution to the correlation diffusion equation for a BFI[(r,t), ${\mathrm{mm}}^{2}/s$].^34^ To increase sensitivity to the decay of these measured curves, fits were restricted to autocorrelation values >1.05. Optical properties were assumed uniform across all mice; absorption and reduced scattering coefficients at 852 nm were fixed at 0.25 and 9.4/cm, respectively.^51^

To assess CVR, the DCS sensor was secured to the optical window, and the mice were placed in an empty cage. Blood flow was monitored continuously (1 Hz) throughout the duration of the experiment. After a 10-min baseline period, mice were given an intraperitoneal injection of 90  mg/kg ACZ (Sigma-Aldrich, St. Louis, MO). ACZ was dissolved in a phosphate-buffered saline solution (Lonza Walkersville Inc., MD), pH 8.8, to yield a 32-mg/mL stock solution. Preliminary results suggested the hemodynamic effects of ACZ peak within 2 to 10 min postinjection and persist for 1 to 2 h (see Fig. S2 in the Supplemental Material). Thus to minimize experiment duration, blood flow was monitored for 10-min post-ACZ injection, after which time the optical sensor was removed, and the animal was returned to its cage.

To quantify CVR, BFI(r,t) was first low-pass filtered at 0.005 Hz. CVR for each source–detector pair was defined as

$$\mathrm{CVR}(r)=\frac{{\left\langle \mathrm{BFI}(r,t)\right\rangle }_{\text{post}}-{\left\langle \mathrm{BFI}(r,t)\right\rangle }_{\mathrm{pre}}}{{\left\langle \mathrm{BFI}(r,t)\right\rangle }_{\mathrm{pre}}}\times 100\%.$$

**Fig. 2:**
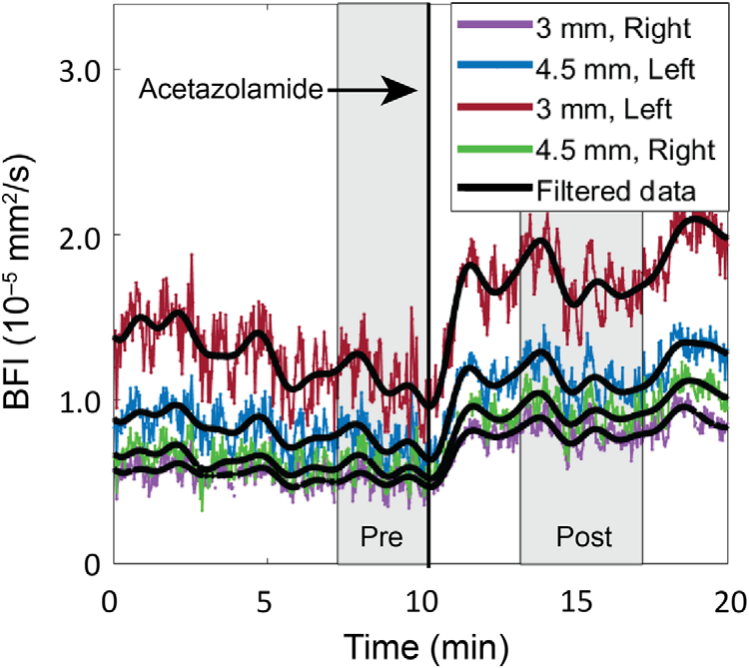
Representative response to Acetazolamide (ACZ). Sample time series of the measured blood flow index (BFI) at the four source–detector separations (colored lines), along with overlaid low-pass filtered time series used for quantification of CVR (black lines). ACZ was given after a 10-min baseline period. Gray shaded rectangles denote averaging windows for the pre- and post-injection periods used for quantification of CVR, which was quantified as the relative percent change in blood flow from pre- to post-ACZ injection.

Here ${\left\langle \mathrm{BFI}(r,t)\right\rangle }_{\mathrm{pre}}$ denotes a 3-min temporal mean of the filtered BFI(r,t) data immediately prior to injection, and ${\left\langle \mathrm{BFI}(r,t)\right\rangle }_{\text{post}}$ denotes a 4-min temporal mean of the filtered BFI(r,t) data that began 2 min after ACZ injection (Fig. 2). We also characterized the response half-time $t_{1/2}$ defined as the time it took the filtered BFI(r,t) data to reach half of the peak response postinjection, i.e., when $\mathrm{BFI}(r,t)=\frac{1}{2}{\left\langle \mathrm{BFI}(r,t)\right\rangle }_{\mathrm{pre}}+\frac{1}{2}{\left\langle \mathrm{BFI}(r,t)\right\rangle }_{\text{post}}$.

### 2.3 Statistical Analysis

Paired Wilcoxon signed-rank tests were used to determine whether baseline BFI, CVR, and $t_{1/2}$ were different between hemispheres (right versus left) and between source–detector separations (3 versus 4.5 mm). Further, linear regression analysis was used to quantify the relationship of BFI, CVR, and $t_{1/2}$ across source–detector separations and hemispheres. Results are reported as slope and intercept [with associated confidence intervals (CI)], along with the coefficient of determination, $R^{2}$. Global estimates of baseline BFI, CVR, and $t_{1/2}$ were calculated by averaging each measured parameter across all source–detector pairs. To assess the amount of variation between mice, we first calculated the mean and standard deviation of each measured global parameter across all mice on each day, allowing us to calculate a daily intermouse coefficient of variation (${\mathrm{COV}}_{\text{byday}}$, standard deviation/mean of parameter X). We then estimated a mean(std) intermouse COV using the ${\mathrm{COV}}_{\text{byday}}$ values (standard deviation/mean of ${\mathrm{COV}}_{\text{byday}}$). Similarly, to assess the amount of variation for a given mouse across days, we first calculated the mean and standard deviation of each measured global parameter for each mouse across all days, allowing us to calculate a COV per mouse (${\mathrm{COV}}_{\text{bymouse}}$, standard deviation/mean of parameter X). We then estimated a mean(std) intramouse COV using the ${\mathrm{COV}}_{\text{bymouse}}$ values (standard deviation/mean of ${\mathrm{COV}}_{\text{bymouse}}$). All statistical analyses were performed in MATLAB.^52^ Hypotheses tests and associated p-values were two-sided. Statistical significance was declared for p-values<0.05.

## 3 Results

A total of 25 measurements were made in 5 mice across 5 consecutive days. Five measurements were discarded: two were discarded due to abrupt movement during intraperitoneal injections, and three were discarded in mouse 4 due to poor sensor contact with the optical window as a result of dried dental cement on the window surface.

Resting state blood flow showed significant temporal variation over the 10-min baseline period, as seen in the representative timeseries in Fig. 2. This temporal variability was not thought to be due to motion artifact, as it was observed even when the animal was at rest.

**Fig. 3:**
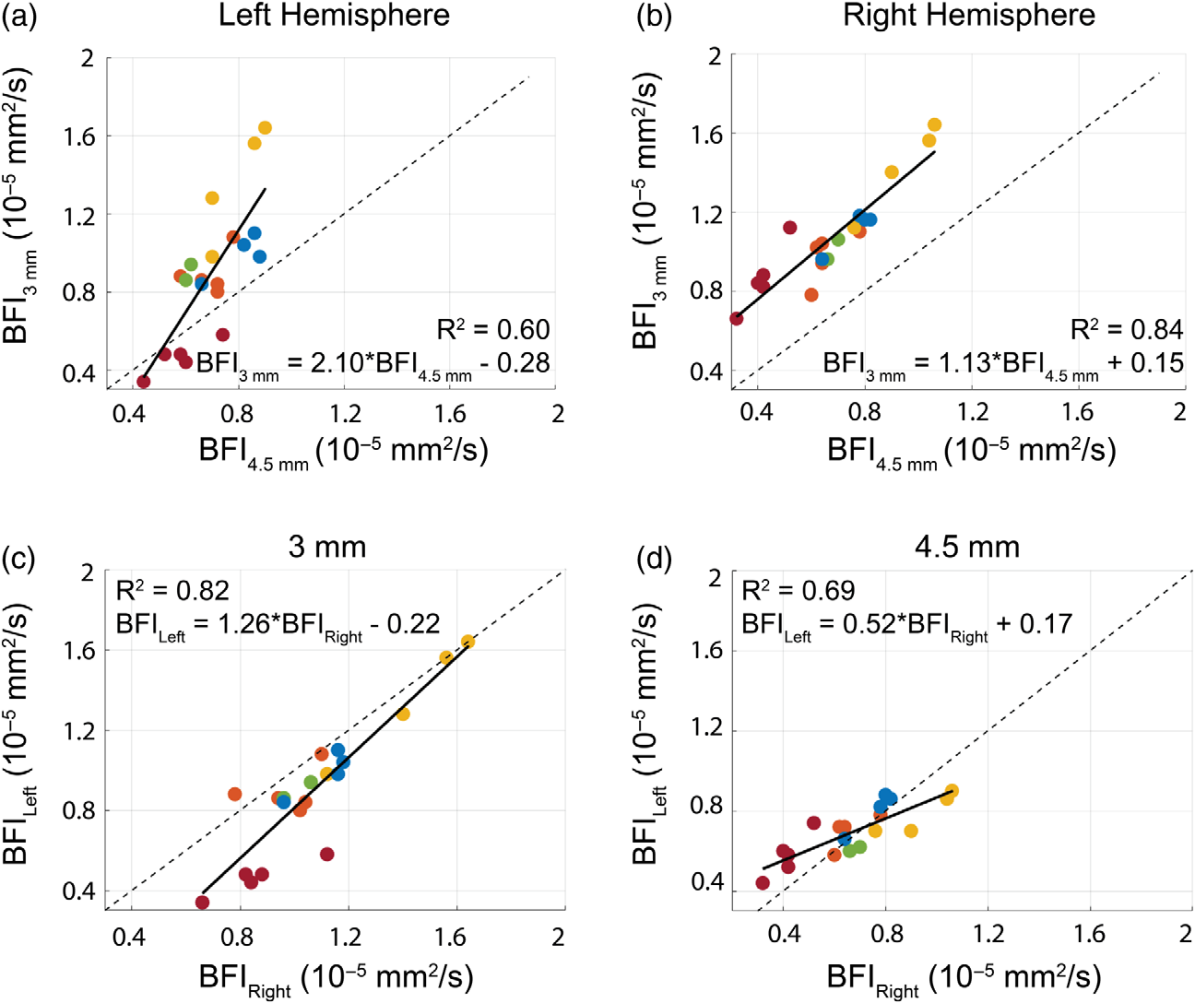
Resting-state BFI. Resting-state BFI at 3 versus 4.5 mm (${\mathrm{BFI}}_{3\;\mathrm{mm}}$ and ${\mathrm{BFI}}_{4.5\;\mathrm{mm}}$, respectively, in units of $10^{-5}\;{\mathrm{mm}}^{2}/s$) across all days, color coded by mouse, for (a) the left and (b) right hemispheres. Right versus left BFI (${\mathrm{BFI}}_{\text{right}}$ and ${\mathrm{BFI}}_{\text{left}}$, respectively) across all days, color coded by mouse, for the (c) 3 mm and (d) 4.5 mm source–detector separations. The solid line in all subplots denotes the best-fit line to the data, whereas the dotted line reflects the line of unity.

**Fig. 4:**
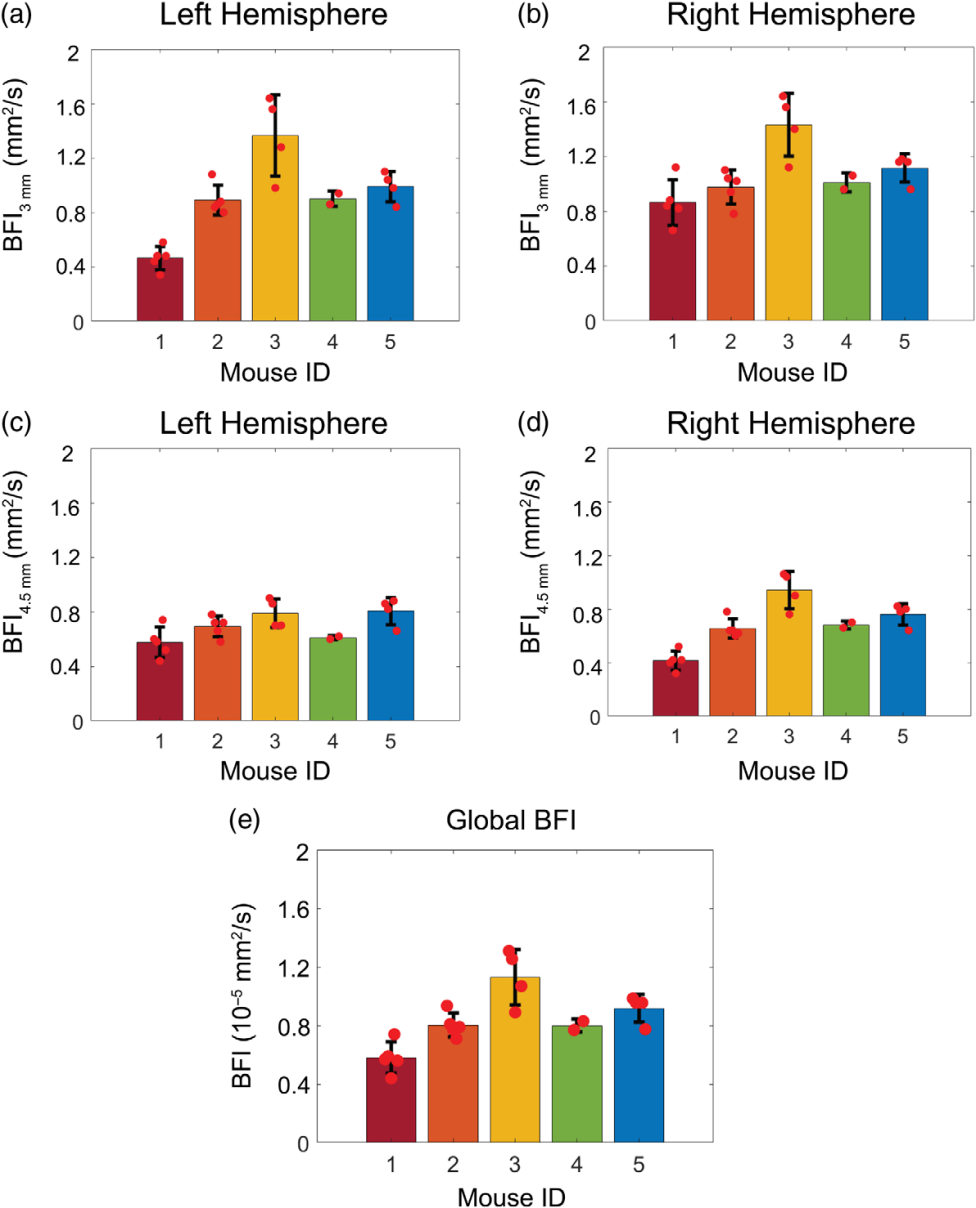
Resting state BFI: (a)–(d) barplot of the mean baseline BFI values for each mouse at each detector and (e) barplot of the mean global baseline BFI values for each mouse. Red dots denote daily values and the black bars denote the standard deviation in the mean across all days measured.

Across all mice, baseline BFI at the 3-mm source–detector separation (${\mathrm{BFI}}_{3\;\mathrm{mm}}$) was systematically higher than BFI at the 4.5-mm separation (${\mathrm{BFI}}_{4.5\;\mathrm{mm}}$) for both the right and left hemispheres (p<0.001 and p=0.02, respectively). At the 3-mm separation, baseline BFI on the left hemisphere (${\mathrm{BFI}}_{\text{left}}$) trended higher than BFI on the right hemisphere (${\mathrm{BFI}}_{\text{right}}$, p=0.06), whereas at the 4.5-mm separation, ${\mathrm{BFI}}_{\text{right}}$ trended higher than ${\mathrm{BFI}}_{\text{left}}$ (p=0.08). Baseline ${\mathrm{BFI}}_{3\;\mathrm{mm}}$ was weakly correlated with ${\mathrm{BFI}}_{4.5\;\mathrm{mm}}$ on the left hemisphere [Fig. 3(a), Table 1] and strongly correlated with ${\mathrm{BFI}}_{4.5\;\mathrm{mm}}$ on the right hemisphere [Fig. 3(b), Table 1]. Similarly, ${\mathrm{BFI}}_{\text{right}}$ and ${\mathrm{BFI}}_{\text{left}}$ were significantly correlated at the 3-mm separation [Fig. 3(c), Table 1] and at the 4.5-mm separation [Fig. 3(d), Table 1]. Intramouse COV of global baseline BFI was 12(5)%, whereas intermouse COV was 26(7)% (Fig. 4, Table 2).

**Table 1:** Summary of correlations between detectors. Results of linear regression analysis quantifying the relationship of BFI, CVR, and $t_{1/2}$ across source–detector separations and hemispheres. Results are reported as slope and intercept [with associated confidence intervals (CI)], along with the coefficient of determination, $R^{2}$, and p-value.

		Slope (CI)	Intercept (CI)	$R^{2}$	p-value
BFI	Left, 3 mm versus 4.5 mm	2.10 (1.91, 2.29)	−0.28$(-0.35,-0.22)\times 10^{-5}$	0.60	0.07
	Right, 3 mm versus 4.5 mm	1.13 (1.08, 1.18)	0.15 $(0.14,0.17)\times 10^{-5}$	0.84	0.001
	3 mm, left versus right	1.26 (1.19, 1.32)	−0.22$(-0.26,-0.19)\times 10^{-5}$	0.82	0.001
	4.5 mm, left versus right	0.52 (0.48, 0.56)	0.17 $(0.16,0.19)\times 10^{-5}$	0.69	<0.001
CVR	3 mm versus 4.5 mm	1.00 (1.00, 1.02)	−0.08(−2.74,2.56)	0.99	<0.001
	Left versus Right	1.00 (0.99, 1.01)	−1.48(−2.18,−0.82)	0.99	<0.001
$t_{1/2}$	3 mm versus 4.5 mm	0.90 (0.88, 0.92)	0.87 (−0.07,1.81)	0.97	<0.001
	Left versus right	0.99 (0.98, 0.99)	1.97 (1.60, 2.34)	0.99	<0.001

**Table 2:** Measurement repeatability. Mean ± standard deviation coefficient of variation within mice (${\mathrm{COV}}_{\text{intra}}$) and between mice (${\mathrm{COV}}_{\text{inter}}$) for global measurements of baseline BFI, CVR, and response half-times ($t_{\frac{1}{2}}$).

	Baseline BFI (%)	CVR (%)	$t_{\frac{1}{2}}$ (%)
${\mathrm{COV}}_{\text{intra}}$	12±5	15±9	24±15
${\mathrm{COV}}_{\text{inter}}$	26±7	19±10	27±11

**Fig. 5:**
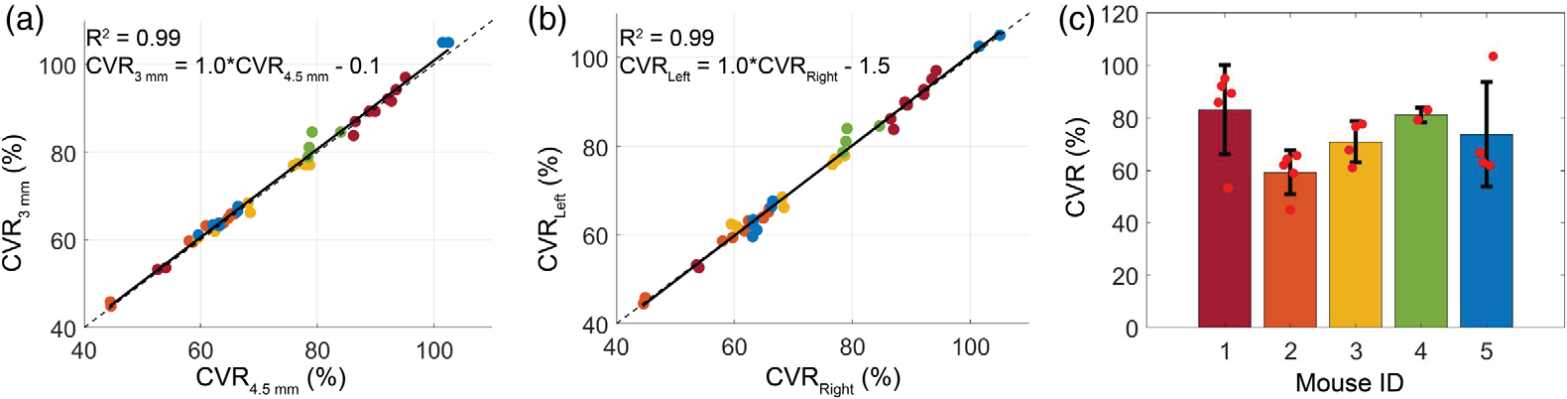
Cerebrovascular reactivity. (a) CVR at 3 mm versus 4.5 mm (${\mathrm{CVR}}_{3\;\mathrm{mm}}$ and ${\mathrm{CVR}}_{4.5\;\mathrm{mm}}$, respectively) across all days and both hemispheres, color coded by mouse. (b) CVR in the left versus right hemispheres (${\mathrm{CVR}}_{\text{left}}$ and ${\mathrm{CVR}}_{\text{right}}$, respectively) across all days and both source–detector separations, color coded by mouse. In both (a) and (b), the solid line in all subplots denotes the best-fit line to the data, whereas the dotted line reflects the line of unity. (c) Barplot of the mean global CVR for each mouse. Red dots denote daily values, and the black bars denote the standard deviation in the mean across days.

Although resting-state BFI differed significantly between source–detector separations and between hemispheres, CVR was remarkably similar (p>0.05 for all paired comparisons). CVR was strongly correlated across source–detector separations [Fig. 5(a), Table 1]. Similarly, strong correlation was observed across hemispheres [Fig. 5(b), Table 1]. Mean(std) global CVR across all mice and all days of study was 73(15)%. The mean(std) intramouse COV of global CVR was 15(9.0)%, whereas the intermouse COV was 19(10)% [Fig. 5(c), Table 2].

**Fig. 6:**
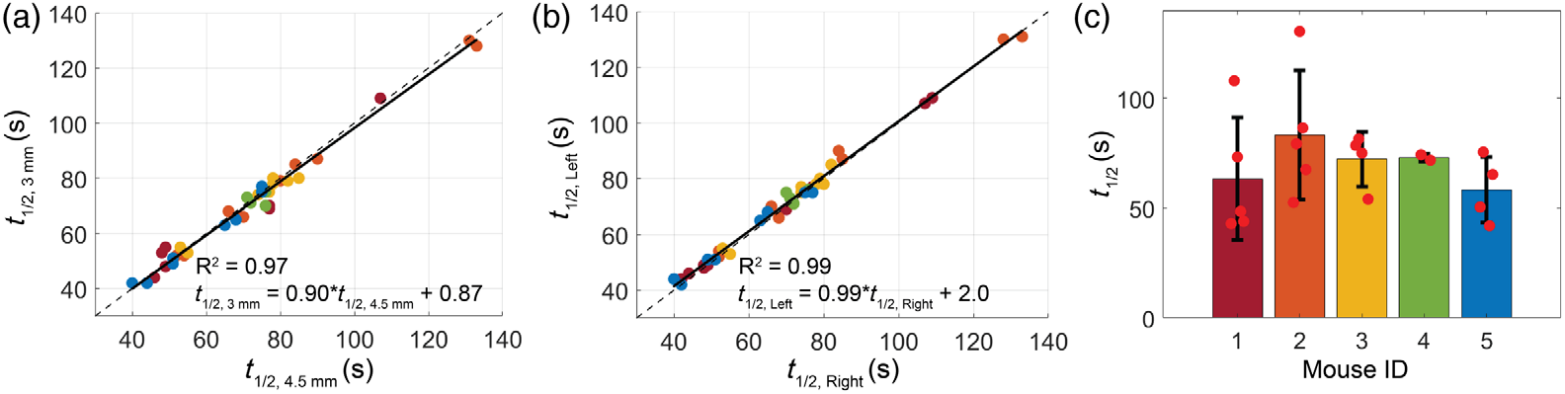
Response half-time. (a) ACZ response half time at 3 mm versus 4.5 mm ($t_{1/2,3\;\mathrm{mm}}$ and $t_{1/2,4.5\;\mathrm{mm}}$, respectively) across all days and both hemispheres, color coded by mouse. (b) Response half-time in the left versus right hemispheres ($t_{1/2,\text{left}}$ and $t_{1/2,\text{right}}$, respectively) across all days and both source–detector separations, color coded by mouse. In both (a) and (b), the solid line in all subplots denotes the best-fit line to the data, whereas the dotted line reflects the line of unity. (c) Bar plot of the mean global response time for each mouse. Red dots denote daily values and the black bar denotes the standard deviation in the mean across days.

Similar to CVR, the response half time ($t_{1/2}$) to ACZ was also highly correlated across source–detector separations [Fig. 6(a), Table 1] and hemispheres [Fig. 6(b), Table 1]. Mean(std) global $t_{1/2}$ across all mice and all days of study was 70(22) s. Mean(std) intramouse COV for global $t_{1/2}$ was 24(15)%, whereas intermouse COV was 27(11)% [Fig. 6(c), Table 2].

## 4 Discussion

Herein, we present an approach to estimate CVR in awake, free-behaving mice via intraperitoneal injection of ACZ coupled with minimally invasive assessment of cerebral blood flow with DCS. ACZ acts to inhibit carbonic anhydrase, an enzyme that aids in the transport of carbon dioxide from the tissues to the lungs. This inhibition increases local carbon dioxide levels by slowing carbon dioxide transport from the tissue to the blood stream, causing an increase in blood flow.^53^ Although intravenous ACZ is commonly used in humans to assess CVR,^54^ it is less common in mice due to difficulties with drug administration (i.e., tail vein injections require restraint acclimation, multiple injections for longitudinal studies create a risk for infection or vein collapse). Herein, we utilize intraperitoneal injection of ACZ to overcome these difficulties. Although drug uptake is slower and sometimes more variable with intraperitoneal versus intravenous injection,^55^ the relative ease of administration provides an obvious advantage.

To characterize this new technique, we quantified CVR in multiple mice across several days. We found that CVR and the response half-time were remarkably similar across hemispheres and across source–detector separations (Figs. 3 and 5), suggesting a homogenous, whole-brain response to ACZ. The homogeneity of this response in mice is in contrast to previous results in humans that show regional differences in brain response to ACZ.^56^ We also characterized the inter- and intramouse CVR response (15% and 19%, respectively, Fig. 4). For context, CVR impairments >25% have been reported for humans with internal carotid artery occlusions,^59^^,^^60^ white matter disease,^60^^,^^61^ stroke,^9^^,^^60^ and traumatic brain injury.^23^^,^^62^ Given the reported variability of our method, we should be sensitive to impairments of this magnitude. Our mean intersubject COV agrees with previous results in mice^57^ and is notably less than intersubject variability seen in humans with ACZ.^58^ We note that one source of variability with this approach is the use of intraperitoneal injection, which entails hepatic metabolism before ACZ enters the systemic circulation and reaches the brain. Differences in metabolism both between mice and across days may cause variability in ACZ response.^55^ In sum, we found this method of measuring CVR to be repeatable without the risks associated with multiple tail vein injections or the confounding influences of anesthesia.

This approach also enabled us to characterize resting-state blood flow in awake, free-behaving mice. Significant temporal variability (∼20%) was observed during the 10-min baseline period that was likely physiological in origin, as motion artifacts were minimal and fluctuations were observed even during periods of inactivity. These variations may be attributed to uncontrolled fluctuations in neural activity, blood pressure, cardiac pulsatility, and/or carbon dioxide tension as the mouse ambulates freely.^63^ With the recent development of high-speed software correlators,^64^ future work may incorporate high-speed (>100  Hz) DCS acquisition to visualize pulsatile flow and better understand these temporal variations.

As expected, we found that the mean resting state blood flow was systematically lower at 4.5-mm than 3-mm source–detector separations given the higher fraction of detected photons at the 4.5-mm separation that penetrate to the deeper, less-vascularized white matter wherein perfusion is lower.^65^^,^^66^ Interestingly, we also observed small, non-significant hemispheric differences in resting state blood flow. The cause of this hemispheric asymmetry is unknown, but regional perfusion asymmetries have also been observed in humans,^67^^,^^68^ and we have also observed similar asymmetries in unpublished results from other strains of mice measured in our lab. Average resting state global blood flow was relatively stable across multiple days of measurement [mean(std) intramouse COV=12(5)%]. However, substantial variation was observed across mice, in agreement with previous publications.^69^^,^^70^ Intramouse variation may arise, to some extent, because we have assumed fixed values for the absorption and reduced scattering coefficient across all mice in our estimation of BFI. Assuming ∼10% variation in absorption and reduced scattering coefficients across mice,^51^ fixing these coefficients to assumed values may lead to errors in BFI on the order of ∼10%.^71^ Future work would benefit from simultaneous estimation of BFI and tissue optical properties.

Limitations of this method include a somewhat time-intensive surgery (∼45  min) and recovery/acclimation period (∼3 days), along with administration of a vasoactive substance that increases cerebral perfusion for up to 2 h postinjection (Video S1). We also note that repeated ACZ administration has been documented to increase production of carbonic anhydrase.^72^ This increase may cause mice to develop drug tolerance and thus may artificially attenuate the CBF response over time. However, we did not observe a decreased response to ACZ over the 1-week time frame of our study. An additional limitation of this method is the assumption that tissue optical properties (i.e., the absorption and reduced scattering coefficients) do not change appreciably in response to ACZ. Recent work in rats has shown that optical properties do not change during hypercapnia;^73^ however, the effect of ACZ on optical property changes is unknown. Given that ACZ and hypercapnia act via similar mechanisms, i.e., to increase carbon dioxide tension in the tissue, we believe this assumption to be a suitable first-order approximation. If false, a 10% increase in the reduced scattering coefficient could lead to errors in rCBF of ∼20%.^44^ Thus as mentioned above, future studies would benefit greatly from the use of frequency domain near-infrared spectroscopy^51^ or multidistance DCS^74^ to continuously quantify absorption and reduced scattering coefficients.

Finally, we comment on the relatively high rate that data were discarded in this study (20%). Three out of five discarded datasets were rejected from a single mouse due to poor sensor contact with the optical window as a result of dried dental dement on the window surface. Future studies should employ careful visual inspection of the window surface during surgery to remove any surface imperfections that prohibit proper sensor contact with the window. The remaining two discarded datasets were rejected due to abrupt movement during ACZ injection that caused the needle (and thus the ACZ solution) to become displaced from its intended target of the intraperitoneal cavity. This motion occurred in two separate mice toward the end of the study (days 4 and 5) and was possibly caused by skin soreness after multiple days of repeated injections. If so, a topical analgesic may mitigate this issue when repeated injections are warranted.

## 5 Conclusions

This study reports a method for longitudinal assessments of cerebral blood flow and CVR in awake, free-behaving mice which can be easily adapted to assess cerebrovascular impairment in various disease states. This approach overcomes several key challenges of vascular reactivity assessment in mice by removing the need for a repeated tail vein injection, femoral arterial catheter, intubation, or the confounding influence of anesthesia. Future work can utilize this method to screen for cerebrovascular impairments in murine models of disease and to track changes in vascular reactivity with therapeutic intervention.
