# Supplementary figures and images for: Cerebrovascular reactivity measured in awake mice using diffuse correlation spectroscopy
Source: Neurophotonics. 2021 Mar 1;8(1):015007. doi: 10.1117/1.NPh.8.1.015007 (PMC7920384; doi:10.1117/1.NPh.8.1.015007)

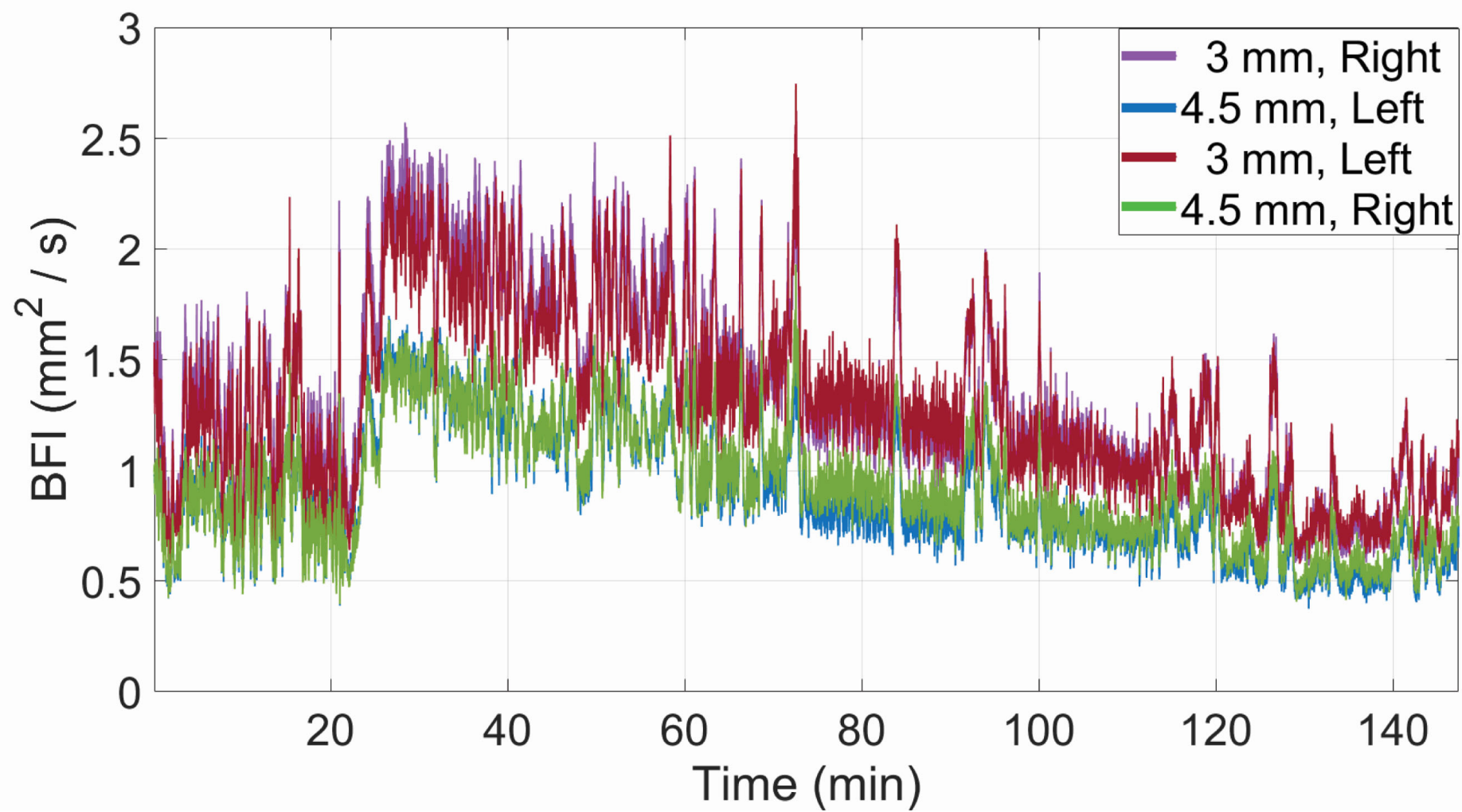

Supplement: Supplementary file 1 [file NPh_008_015007_SD001.pdf]
